# Muslim immigrant women’s views on cervical cancer screening and HPV self-sampling in Ontario, Canada

**DOI:** 10.1186/s12889-016-3564-1

**Published:** 2016-08-24

**Authors:** Mandana Vahabi, Aisha Lofters

**Affiliations:** 1 Faculty of Community Services, Daphne Cockwell School of Nursing, Ryerson University, Toronto, ON Canada; 2 Centre Global Health and Health Equity, Ryerson University, Toronto, ON Canada; 3 Graduate Program in Immigration and Settlement Studies, Ryerson University, Toronto, ON Canada; 4 Centre for Research on Inner City Health, Li Ka Shing Knowledge Institute, St. Michael’s Hospital, Toronto, ON Canada; 5 Department of Family and Community Medicine, University of Toronto, Toronto, ON Canada; 6 Department of Family and Community Medicine, St. Michael Hospital, Toronto, ON Canada; 7 Institute for Clinical Evaluative Sciences, Toronto, ON Canada

**Keywords:** Cervical cancer, Screening, HPV self-sampling, Muslim women, Immigrants, Attitudes and perception (or appropriateness and acceptability)

## Abstract

**Background:**

Canada has observed significant decreases in incidence and mortality of cervical cancer in recent decades, and this has been attributed to appropriate screening (i.e., the Pap test). However, certain subgroups including Muslim immigrants show higher rates of cervical cancer mortality despite their lower incidence. Low levels of screening have been attributed to such barriers as lack of a family physician, inconvenient clinic hours, having a male physician, and cultural barriers (e.g., modesty, language). HPV self –sampling helps to alleviate many of these barriers. However, little is known about the acceptability of this evidence-based strategy among Muslim women. This study explored Muslim immigrant women’s views on cervical cancer screening and the acceptability of HPV self-sampling.

**Methods:**

An exploratory community-based mixed methods design was used. A convenience sample of 30 women was recruited over a 3-month period (June–August 2015) in the Greater Toronto Area. All were between 21 and 69 years old, foreign-born, self-identified as Muslim, and had good knowledge of English. Data were collected through focus groups.

**Results:**

This study provides critical insights about the importance of religious and cultural beliefs in shaping the daily and health care experiences of Muslim women and their cancer screening decisions. Our study showed the deterring impact of beliefs and health practices in home countries on Muslim immigrant women’s utilization of screening services. Limited knowledge about cervical cancer and screening guidelines and need for provision of culturally appropriate sexual health information were emphasized. The results revealed that HPV self-sampling provides a favorable alternative model of care to the traditional provider-administered Pap testing for this population.

**Conclusion:**

To enhance Muslim immigrant women screening uptake, efforts should made to increase 1) their knowledge of the Canadian health care system and preventive services at the time of entry to Canada, and 2) access to culturally sensitive education programs, female health professionals, and alternative modes of screening like HPV self-sampling. Health professionals need to take an active role in offering screening during health encounters, be educated about sexual health communication with minority women, and be aware of the detrimental impact of preconceived assumptions about sexual activity of Muslim women.

## Background

Increased efforts to screen for cervical cancer using Pap tests have led to declining mortality rates, especially in developed countries like Canada [1–4]. However, screening rates remain low for certain subgroups of women, including immigrants, placing them at higher risk of advanced cervical cancer and poor health outcomes [5–7]. Among immigrants, there are specific subgroups like Muslim women who encounter further challenges in getting screening for cervical cancer. This is a concern in Canada, especially for provinces like Ontario, where the high proportions of immigrants includes a large and increasing proportion of people of Muslim faith. The number of Muslims in Ontario increased by 65 % from 352,530 (3.1 % of the population) in 2001 to 581,950 (4.6 %) in 2011 [8, 9], and is expected to increase further with the increasing rate of immigration and refugees from Muslim countries. The majority of Muslim immigrants come from South Asian and Middle East countries [8, 9].

Low screening rates have been demonstrated among immigrants, those from low income areas, and older women, with inter-sectionality of these factors leading to much lower rates (31.0 % of those with all three factors vs 70.5 % of those who had none of the factors in one recent Ontario study) [6]. Other Ontario studies that have examined rates of cancer screening by region of origin showed that immigrant women from regions with high Muslim populations (i.e., South Asia, the Middle East and North Africa) had lower rates of cervical and breast cancer screening than their immigrant counterparts [10–12]. Canadian Muslims experience difficulties accessing sexual health information and cancer screening services due to systemic barriers (e.g., lack of a family physician, inconvenient clinic hours, language difficulties), dominant discourses that discourage open dialogue about sexualities within their communities, religious pedagogy and strict regulations regarding modesty, premarital virginity and sexual behaviour [10–21].

Efforts are being made to target under screened groups with new outreach methodologies that bring services closer to the women. Given the strong correlation between cervical cancer and high risk Human Papilloma Virus (HPV), self-sampling for HPV is being considered as an alternative method to promote uptake of cervical cancer screening among under- or never screened women in Canada and other countries [22–27]. The ability to collect one’s own sample in the privacy of one’s home helps to alleviate many of the current barriers faced by immigrant Muslim women and consequently can promote participation in screening. Although literature provides strong evidence of high acceptance and positive attitude of women toward self-administered HPV testing [22–27], there is no information about Muslim immigrant women’s preferences and their acceptance of such a strategy. To address this gap, we explored Muslim immigrant women’ beliefs and attitudes towards cervical cancer screening and their acceptability of HPV self-sampling in the Greater Toronto Area (GTA) of Ontario, Canada. The study was guided by the Population Health Promotion Framework [28].

## Methods

This pilot study used an exploratory community-based sequential mixed-methods design, consisting of a detailed questionnaire followed by focus groups to explore Muslim immigrant women’s knowledge, beliefs and attitudes about cervical cancer and screening, and the cultural relevance, appropriateness and acceptability of self-sampling for HPV. A socio-environmental approach which encompasses the principles of social justice and equity was used to address health disparities [29, 30]. This paper focuses on the findings from the focus group component of the study.

### Sample

The study used a convenience sample of 30 women recruited with collaboration from study community partners (e.g., faith-based facilities-mosques) and snowball sampling. Community-based Research Assistants (RAs) from the Muslim community recruited women for the study based on the following inclusion criteria: (1) were foreign-born and self-identified as Muslim, (2) were aged 21–69 years old in line with provincial cervical screening guidelines [31], and (3) were comfortable communicating in English. The study protocol received ethical approval from the Research Ethics Review Board at Ryerson University (REB 2016-036).

### Data collection and analysis

Data were collected by community-based RAs between May and August 2015. Prior to participating in focus groups, participants completed the study questionnaire that captured information on their socio-demographic characteristics, health status and health care utilization, cervical cancer screening practices and their attitudes towards HPV self-sampling vs. provider-administered sampling. The women participated in one of three focus groups which was offered either in one of the RAs homes or at a faith-based facility (study community partner). The focus groups were facilitated by the authors, conducted in English, and were tape recorded with participants’ permission. Each focus group included ten women and lasted for 2 h. The interview guide included 6 major questions:

1. *What are the experiences of Muslim immigrant people in terms of living and working conditions in Canada?*
2. *What are your beliefs and values about cancer?*
3. *What are your beliefs and values about cervical cancer and screening?*
4. *Have you ever faced any challenges in accessing sexual health, including cervical cancer screening, information and services in Canada?*
5. *What do you think about HPV self-sampling?*
6. *What other approaches would be useful to promote cervical cancer screening uptake among Muslim women (*e.g.*, mobile screening bus, education0 etc.).*

Prior to asking participants’ input regarding HPV self-sampling, the authors provided women with a short demonstration of an HPV self-sampling kit and then circulated a few kits among the participants to allow for a full examination of the instrument.

### Analysis

Data were analyzed using an inductive thematic analysis technique [32]. Analysis involved systematic reading of the text, highlighting important passages and words, and then organizing them into preliminary codes or category schemes. Further interpretation and modification of coding was conducted after rereading the text sorted using the preliminary codes and checking for disconfirming evidence [33, 34]. The integrity of data interpretation was maintained using strategies to ensure credibility (member check, audiotapes), confirmability (inquiry audit), and authenticity (use of direct quotes). Credibility was enhanced through follow-up phone calls to participants to elicit their input about the findings and interpretations [35]. Furthermore, to ensure correct recording of the study participants’ responses we audio-taped focused groups, and transcribed them verbatim (generating 140 pages of data). Confirmability was also ensured though an independent review of the study’s audit trail by an external researcher.

## Results

### Socio-demographic characteristics

Table 1 provides more details about the socio-demographic characteristics of the women. Of the 30 participants, half were from West Asia (Iran) and the rest from South Asia (i.e., Pakistan and India). The participants’ ages ranged from 21 to 61 years with a mean age of 40 years. All the women were either landed immigrants or held Canadian citizenship status. The majority (60 %) had lived in Canada for 10 or more years. Twenty-three percent were new immigrants (i.e., lived in Canada less than 5 years), while 17 % had been there for 5–9 years. Eighty percent of the women were married or in common-law relationships, with the majority having children. The group had a relatively high level of education with 90 % having a college or university education. Over 80 % reported good or excellent English communication. Over 50 % of the women were unemployed or worked part-time. Eighty-three percent of women had either relatives or friends living in Canada. The two ethnic groups were similar in terms of the characteristics presented above.

**Table 1:** Socio demographic Characteristics of Participants

	#
Age - mean = 39.7+ 11.4, range 21–61,	
Age Group	
21–39	14
40–61	16
Ethnic origin	
West Asian (i.e. Iranian)	15
South Asian (i.e. Pakistani/Indian)	15
Length of time in Canada	
0–4 years	7
5–9 years	5
10 or more	18
Immigration status	
Landed immigrant/Canadian Citizen	30
Current relationship status	
Divorced/Separated	2
Married/common law	24
Single, never married	4
Have Children	
Yes	21
No	9
Rating of English reading, writing & speaking abilities	
Excellent	10
Good	15
Fair	5
Highest level of education	
High School (12 grades) or equivalent	3
College (e.g. diploma) or university (e.g. BA, B.Sc.)	16
Post-graduation (e.g.MA, PhD), some or completed	11
Current employment status in Canada	
Full-time employed (Minimum of 35 h/week)	6
Other	7
Part-time employed	7
Unemployed	10
Average # hours worked per week	
20–30 h	2
30–40 h	7
40–50 h	4
Less than 20 h	6
Approximate household annual income from all sources after taxes	
Less than $25,000	3
$25,000–$40,000	3
$41,000–$75,000	6
More than $75,000	4
Don’t know/Don’t want to answer	14
# of people (both children less than 18 years old and adults) living in household	
1	2
2	5
3	10
4	6
5	2
6	1
Have relatives living in Canada	
Yes	25
No	5

Ninety three percent of women aged 21–39 years indicated they would use HPV self-sampling compared to 44 % of older women (40–61 years) (*X*^2^ = 8.10, *p*- value 0.007). Similarly 88 % of younger women (i.e., 21–39 years) were more willing to receive sexual health information and undertake cervical cancer screening compared to 45 % older Muslim women (40–69) (*X*^2^ = 6.94, *p*- value 0.004).

### Muslim immigrant women’s living and working experiences in Canada

Participants reported not only facing challenges that were common to other immigrant groups such as difficulty finding employment, demotion of social class, limited social support, but also specific challenges associated with their religious affiliation and cultural attires like discrimination and social exclusion.

Finding employment was a major issue encountered by the women and their spouses, particularly during the first few years of their settlement in Canada. Lack of Canadian education or Canadian work experience hindered attaining positions which were in par with their education and training. This is reflected in the following excerpts:

“*Actually finding proper jobs has been the most challenging things for me and my husband in Canada. Your qualifications and experiences are not valued regardless of your qualifications, everywhere you go they ask for Canadian experience”.*

*“He [husband] has 20 years of work experience back home but that does not count here”*

*“I’ve been living in Canada for exactly 5 years. My husband, did not pass three times the board exam for physiotherapists but the last one, we are waiting to hear…if he doesn’t pass the last one … then we have to go back because there is no position for him here”.*

Participants expressed a decline in their social status, economic power and social support. Although the majority of the participants reported having a friend or family member in Canada they felt their social support network was not as extensive as back home to provide them the required assistance with the challenges of settlements. Furthermore, a few complained of the commuting cost which limited their mobility both locally and internationally.

*“It is very stressful because I’m working in a store and he [spouse] is working part time and we just managing our day to day expenses”.*

*“I don’t have anybody here, no friends, no family, no experience living outside Iran, I try to find a way to adjust myself. It’s been somewhat challenging for me, you know, because my husband is back home. I am here with my two teenage daughters”.*

*“I am here 10 years and I am far away from my friends and family. I only can visit them every 3 years. It is so expensive going back.”*

Some participants discussed abuses and discrimination which they encountered because of their religious affiliation and cultural attire (hijab- i.e., headscarf) while in Canada. They criticized the public for failing to realize that wearing hijab was a woman’s choice and her autonomous decision which should be respected. The following discriminatory and biased treatments were recounted by participants: request to remove the hijab in workplaces, being denied jobs because of their religion and appearance, and receiving derogatory remarks at work or public places. One participant stated:

“They look at us completely differently because of our hijab you know. Nobody can feel it, we can feel it. The manner in which other people look at me, sometimes it bothers me.”

However, discrimination was also experienced by participants who did not wear the hijab. Not being fluent in English and having accents resulted in women being treated as inferiors. This is reflected clearly in the following statement:

*“I don’t wear a Hijab most of the time, just when I come to the mosque. I have felt this kind of situation [discriminatory attitudes] not because of my hijab but because of my language -- When we talk not very fluent English, they know that we are immigrants. So I feel that somehow they don’t respect us as people, you know? It’s just because you are a foreigner, you are an immigrant, you are from other countries, and [they consider] you as not [being] so wise. It is discrimination not just because of a scarf”.*

### Beliefs and values about cancer

Women’s views of cancer reflected their fear of the disease. They often referred to cancer as a “*death sentence*”. The majority described cancer as a “*deadly disease*”, “*scary”, “painful”, “horrible”,* and/or *“very expensive and time consuming way to death”*. Cultural factors appeared to play a role in how women perceived and responded to cancer. Fear of cancer stemmed from a perceived lack of cure or treatment and observed high fatality for the disease. Most incidences recounted by participants focused on those who they knew and died of cancer and almost none on cancer survival. One participant stated: “*in our culture, whenever we get cancer, we consider it as death sentence whereas in Canada I feel that the emphasis is more on the positive side, on hope that you could do something about it”*.

Some believed that talking or even thinking about cancer could be inviting the disease to their lives. There was limited discussion or sharing of information about cancer among family members and friends. One reason provided was the belief that cancer is hereditary and informing children might scare them, particularly when there is no cure. Hence, most did not know if they had a family history of cancer. One participant stated “*I did not know my grandmother died of breast cancer until we came to Canada and I started having problem with my breast. It was then my mother told me.”*

### Beliefs and knowledge about cervical cancer and screening

The majority of the participants associated cervical cancer with promiscuous sexual behavior and considered it as a sexually transmitted disease. Most believed that Muslim women had lower risk of getting the disease as they did not have multiple partners, and did not engage in premarital sex. One participant stated:

“*I think they [Muslim women] are not immune because there are so many factors, but based on the early age of having sexual life, starting sexual life and multiple partners, I think there should be less percentage [women with cervical cancer] in comparison to western communities. Not totally immune but are at lower risk of cervical cancer”.*

Some of the participants did not know what the risk factors for cervical cancer were, at what age cervical cancer screening should be commenced or what the appropriate screening period was. The following excerpts demonstrate the limited knowledge regarding cervical cancer and screening among the participants:

“*We don’t know what the actual causes are”.*

*“I don’t know about the age but I think after marriage”.*

*“I think that Pap test is recommended every year [overlapping voices “not sure” “every 2 years”, “every 3 years”]*.

“*I don’t know if I remembered this correctly. But she [attending physician] sort of said that -- cervical cancer – because you get your period and stuff, so it’s like cell division? So it can happen because of stuff like that? [Lowered voice indicating she’s not sure]. Yeah I don’t know”.*

Many participants stated that risk factors of cervical cancer included being sexually active at an early age, having multiple partners, having Human Papilloma Virus, and family history of cervical cancer. Other reported risk factors included stress, poor nutrition, physical activity, smoking, being overweight or obese, and exposure to radiation. Furthermore, having a combination of these factors was pointed out as increasing the chance of getting cancer.

In general, the discussion revealed a lack of awareness about cervical cancer screening. Several participants did not know what cervical cancer screening meant as clearly is reflected in the following excerpts:

*“I have a question, what do you mean by screening? Is it a different program than when we go to family doctor and we do a check-up, we do blood testing?”*

*“So my family doctor has to ask me to do this? May be sometimes I’m not at risk so my doctor does not do it. Right?”*

Women stated that issues related to female reproductive organs are not discussed openly as much as other health conditions mainly because of its connection to sexual activity. One participant stated:

“*Since growing up, we are taught to keep our sexual life private and do not discuss it. It is a forbidden subject. Some young women have difficulty with their sexual life but cannot talk to anyone … they think that sex is a dirty thing, nothing to desire. Because of the teaching they received this way growing up.”*

*“I think that if I were going to tell my parents that I’m going for cervical cancer screening, they would be concerned that I have sex and I have AIDS or Cancer.”*

For some women the concept of prevention was a farfetched and unfamiliar idea. They often sought medical care when faced with persistent symptoms that were unresponsive to home remedies. The following excerpt clearly demonstrate this notion:

*“The idea of prevention is not like here, there [India] it’s only if you have a [medical] problem, then you go to get it solved.”*

*“I was raised in Pakistan until I was fifteen. So I never got my physical in those 15 years. You know, it does not seem important – you get vaccinations as a kid and stuff and that’s sort of where it ends. If you have a problem, then you go to the hospital to go see a doctor. But it’s very rare for you to have yearly check-ups where you are being told that you have to should go for screenings and stuff like that.”*

*“In our country [Iran] we pay for everything, doctors’ visits, medication, tests. We go to see doctors when we have problem not when there is nothing to discuss.”*

Women’s expressed the importance of receiving information and recommendation from their attending physicians regarding uptake of cancer screening in Canada. The majority considered physicians as trusted and authoritative figures whose recommendations they would follow. If their doctor did not recommend a test then it meant to them that the test was not necessary

*“I only went one time. She hasn’t recommended it. So I think I don’t need it right now.”*

*“We’ve been here two and a half years. And I had difficulty in the initial year that I was here because I didn’t know the system. I had my family doctor but I did not have any tests or things done on me.”*

*“I don’t recall when my doctor did a physical check-up or pap-test on me. I see her when I have health problems or take my children there but she has never discussed anything other than what I am there for.”*

### Challenges accessing health and sexual health services in Canada (including cervical cancer screening)

Muslim women’s discussion of challenges in accessing health services revealed a number of individual and structural barriers. These included 1) lack of knowledge of the Canadian health care system, 2) difficult accessing female physicians, 3) language and ethnic match/mismatch in health encounters, 4) long wait time and enforced prioritization of health concerns, and 5) limited access to transportation and time constraints.

#### Lack of knowledge about the Canadian health care system

A common challenge, especially during the initial years in Canada, was the lack of knowledge about the Canadian health care system. Many women were not aware of how to find a physician, the referral process to see a specialist, or the availability and locations of health services. Both ethnic groups indicated that the health care system in their home countries was based on fee for service which was remunerated by the patients. This gave people the autonomy for self-referral to specialists. Although they were aware of the Canadian universal health care system at the time of the entry to Canada, they had no knowledge of the health services that were available to them and how to access them. They stated that they did not know about the role general physicians played as gatekeepers for accessing specialists or other health services.

*“In our country we go to specialist directly but everything has to go through the G.P here [Canada]. It takes time to get the referral and see a specialist. It took me like more than four months to see an Orthopedic”.*

The majority of women had difficulty finding a family physician when entering Canada and for some it took several years before securing a permanent family physician. Most got referrals from friends or family and often had to go through multiple steps before they got a family doctor. Use of the government *Health Care Connect program*, a program meant to link unattached patients to a family physician, to get matched with a physician in their location was often associated with a very long wait times. Failure to access family physicians forced some of the participants to resort to medicating themselves or their children with antibiotics and other drugs which they brought with them from their home countries. The following quotes express this challenge:

*“One of my primary experiences was when I was trying to find a family doctor for myself and my family. So the government has this program (Health Care Connect program) where you can sign up your family and they will find a family doctor for you that is looking for patients. So they match you with somebody. But it took a while, it took about a year or so before they matched us with a family doctor but then our family doctor kept changing. Every year we wouldn’t get a physical done because the doctor would change and then the transition would get lost. So I think in terms of access, finding a family doctor is a primary concern”*

“*For an immigrant there are lots of things that you don’t have enough information and you need someone to help you, and fortunately I have friends and family here and ask them to help me, and I chose my family doctor by their recommendation. But if they weren’t here, I think maybe I had a lot of problems, because we are not familiar with this system, it takes time to know how you can do many things”.*

*“I’ve seen other people bring antibiotics when they come here initially. But I’m sure when they have good family physicians, then they don’t have to.”*

Women expressed the need for more information about how they could access physicians and other health services.

#### Access to female physicians

The majority of participants did not feel comfortable having a male physician due to religious and cultural beliefs. However, accessing a female family physician or specialist was reported to be quite challenging. Although several women had female physicians which they often found through family or friends, they were often referred to male specialists (e.g., gynecologists). Getting access to female specialists was often associated with long waits (up to six months) or having to travel long distances to specialists in other cities. Most participants felt that the Canadian culture of requiring a referral from an attending physician to see a specialist further lengthened the wait time.

“*I think it is very important especially for a Muslim woman to have a woman doctor because of religious beliefs– they are more comfortable with woman family doctor”.*

*“Health Care Connect program only ask for location preference, where you live, and then they will try to match in your area. You couldn’t set up for any other preferences. We usually have to find female doctors through friends and family.”*

*“All the Muslim women have concerns about seeing the lady doctor. It is easier to talk about our gynecologist problems or our breast problems, or other problems. My family doctor is a lady doctor. But when I go and tell them, I have to wait for a long line up. For my gynecologist, I wait for six months. Yeah because I need a lady doctor and she doesn’t have the time -- the doctor I want to see. That’s the problem.”*

#### Language and ethnic match/mismatch in health encounters

Several participants indicated preference for having physicians who spoke their language and were from their own ethnic groups. They remarked that it was relatively easy to explain common conditions like colds or dizziness in English but more difficult to do so for complex and sensitive issues like female problems. They felt sharing a similar cultural background would allow physicians to better relate to women’s health concerns. This is expressed in the following quote:

“*There are GP’s who, I guess there are less culturally sensitive. I guess that’s what it is – we go to the doctor with our moms, and I’m not married and I’m not comfortable when my GP says “are you sexually active?” and my mom is sitting beside me. No I’m not!! (Laughs). So, that’s why it’s nice for a lot of us to choose a GP that’s are from our own culture because they won’t ask questions like, “are you sexually active?”*

*“Because of language, it’s hard for me to explain a heart problem. Catching a cold it’s easy. You have fever and some kind of dizziness, it’s easy but the female problems are hard for me to explain the situation. So I prefer someone who could speak my language.”*

Although most women preferred to have physicians with similar ethnic and linguistic background, they felt that having a physician from the same culture did not always guarantee access to sexual health services or information. Some stated that physicians with similar ethnicity often avoided asking them about their sexual activities or testing them for sexually related diseases such as cervical cancer. They felt it was mainly either due to physicians’ cultural upbringing which encouraged abstaining from discussion related to sexuality or their medical training that placed more focus on treatment rather than prevention of the disease. Younger and unmarried participants also expressed concern about not being asked by their physician whether or not they are sexually active. They explained that preconceived assumptions about Muslim women’s sexual life automatically ceased discussion about sexual health during health encounters. Their hijab and the fact of being a Muslim woman automatically made their physician assume that they were abstaining from any sort of sexual activities prior to marriage. As indicated in the following quotes:

*“I know I’m wearing hijab but maybe I am sexually active so they should not assume.”*

*“But her doctor is from Middle East and never did a pap-test on her until when her son was born. He is 6 years old. Only in 9 years that she has been here, she only had one Pap smear. And never told her about the yearly checkup or anything*”

“*Sometimes I think I have to explain and ask my family doctor to do something, −- I don’t want to say that she didn’t care. She knows her job, but I have to ask “is it better to have some kind of examination?”*

#### Long wait time and enforced prioritization of health concerns

Several participants criticized long wait times, often more than an hour, to see their attending physician, even when they had made the appointment ahead of time. In addition, some of the participants stated that they were expected to only present one health problem per visit and to book appointments accordingly for any additional problems. This is reflected in the following excerpt:

*“I think every three months I go to emergency unit because it is so hard to see your own doctor. Many people do the same”.*

*“It is hard to see your doctor even when you have an appointment. For example, I have an appointment at 10 o’clock and at 10 to 10 we are there. But we should wait, maybe for one hour, or one and a half hours, or even more”.*

*“You cannot ask more than 1 question per visit. They have a sign in waiting room saying this.”*

*“That happens to me always, we had appointment at 10 am and went inside at 1 pm. We had an appointment at 1 pm and went inside at 4 or 5. And after all that waiting you are only allowed to only ask not more than 2 questions per visit.”*

#### Access to transportation and time constraints

Lack of transportation was a deterrent to seeking medical care. This was worsened by cold weather and long travel distances, especially in the suburbs. The cost of commuting as well as the lost pay due to time off to make the appointment was highlighted as a major barrier. Some women worked during the week and their work hours overlapped with their physicians’ office hours. This required taking time off from work to visit their physicians or using alternative options such as walk in clinic or ER visits which did not ensure continuity of care.

“Transportation is not easy especially during the cold weather. Also if you don’t have family/friend to give you a ride or babysit for you then you have to pay. Not to mention taking time off from work. So it’s hard to see your doctor under these conditions”.

### Views about HPV self-sampling

Both advantages and disadvantages of using HPV self- sampling were identified. Many women expressed positive attitudes towards using HPV self-sampling and were willing to try it out. One of the advantages of this approach was its consideration for Muslim women’s modesty and privacy. Women stated that they could do the test in the privacy of their home and mail it without even family members being aware. In addition, they considered it to be cost-effective and saving time since they did not have to take time off work, or pay for childcare or transportation. An interesting aspect that emerged was the benefit of this method for undertaking screening among Muslim women who were sexually active prior to marriage. Pre-marital sexual activity is a cultural taboo in Muslim communities with severe consequences that can include being disowned by one’s parents. Hence, this approach would allow sexually active unmarried Muslim women the opportunity to participate in cervical cancer screening without their families’ knowledge.

The disadvantage of using HPV self-sampling was related mainly to women’s ability to accurately take the sample. Some women had concerns about their own abilities and skills in taking the sample. However, the majority felt this barrier could be overcome by enhancing women’s self-efficacy in conducting the procedure through clear instructions. A clear guide, in simple language with instructions on how to conduct the test, in one’s native language with a stamped return envelope was highly recommended. Women also emphasized the importance of providing an explanation as why the test was important in relation to cervical cancer.

Another issue which was raised by women was the cost associated with HPV self-sampling, since it is not currently part of Canadian cervical cancer guidelines. Women stated that if they have to pay it out of their own pocket then the cost becomes an important factor in undertaking the test. Although a cost of $30 or less appeared to be reasonable for the majority of the participants who preferred this method, not paying for it was the best option.

It is interesting to note that HPV self-sampling preference varied by age. The younger women were more willing to undertake the test compared to older women. This appeared to be related to older women feeling less confident in conducting this procedure accurately compared to younger women. It was also noted that younger Muslim women were more open to receiving sexual health information and undertaking cervical cancer screening as opposed to older Muslim women. This view is demonstrated in the following quote:

*“If we look at ourselves, me and some of us are not married and have not done the Pap test but we still cared enough to come and to educate ourselves. So I feel like there’s such a huge variety of people that some might and will be interested to do it. So I feel like it is very subjective but in my opinion, the resource should be there and our women should be encouraged to use it. The culture has to change. It can be changed through holding these kind of focus groups that you are holding and it’s done through daughters explaining to their parents the importance cancer screening.”*

### Views regarding other approaches (like mobile screening) to promote cervical cancer screening among Muslim women

Although women reflected mainly on the use of HPV self-sampling, alternative strategies such as the utilization of a mobile bus in promoting cervical cancer screening among Muslim women were also discussed. This method involves providing the Pap test in a mobile bus at a community location accessible to women.

There were mixed views about the use of mobile screening. One participant who had used the mobile bus stated that despite her initial skepticism she had a positive experience. She found having the Pap-test by a female health care provider in the bus was as comfortable as having it in a physician’s office. However, for some women the mobile bus carried negative connotations. The mobile bus was associated with not being as clean or hygienic as a stationary hospital, especially for tests like Pap tests where higher standards of cleanliness were desired. They were also worried about lack of privacy and bringing attention to themselves by using the mobile bus. The majority of women indicated that mobile bus may be appropriate for simple blood tests or vaccination but not for conducting pap-test which is associated with exposure of private parts.

#### Raising awareness

The dialogue disclosed that the provision of cancer screening services although necessary was not sufficient for uptake. The majority commented that, unlike breast cancer, Muslim women were not aware of cervical cancer and thus not likely to go for screening. The importance of raising awareness was stressed and provision of culturally and linguistically appropriate information about the importance and necessity to undergo screening was highlighted. Several formats for dissemination of information were suggested, such as one-on-one consultation with a female health care provider and small women-only workshops led by a trusted member of the community/medically trained source e.g., female doctors or nurses. A big motivation for women to participate in the workshops would be if they were informed that the information could also benefit their family, particularly their children. Some participants felt that women who participate in the one-on-one consultation or workshops could share the information gained with their daughters. The inclusion of Muslim women as equal partners in decisions related to their health and empowering them with knowledge were considered to be highly important and a potential facilitator for uptake of cervical cancer screening.

## Discussion

In this study we held focus groups with 30 Muslim immigrant women in the Greater Toronto Area to explore their working and living experiences in Canada, their beliefs and values about cancer in general and cervical cancer specifically, challenges they experienced in accessing sexual health information and services, and their views about using HPV self-sampling method and other potential strategies in promoting uptake of cervical cancer screening. The majority of women reported that they would be willing to try HPV self-sampling and would prefer this method to current provider-administered sampling methods. Barriers to self-sampling included confidence in the ability to perform the test, perceived cost of HPV self-sampling, and facilitators included cost saving, convenience and preserved privacy. The study provided further elucidation and new insights on the role of religion and cultural beliefs about cervical cancer and in undertaking cervical cancer screening.

Our study found that Muslim immigrant women like other immigrant groups in Canada face several challenges during their settlement like difficulty findings jobs despite being highly educated and skilled, experiencing decline in social class, having limited social support, language difficulties, and lack/limited knowledge about the Canadian health care system and resources [10–20, 36–38]. However, Muslim women wearing the hijab faced additional challenges of tangible discrimination and abuse in employment and public spaces. This supports previous findings highlighting the unique forms of discrimination that Muslim immigrant women face at the intersection of religion, race and gender [39, 40]. The stereotypical beliefs about Muslims being sympathetic to terrorism and hence potential threats to national security as well as the hijab being viewed as a symbol of woman’s subservience to male oppression, and a sign of docility and powerlessness can contribute to Muslim women’s feeling of alienation and exclusion. These challenges taken together may greatly influence Muslim immigrant women’s health concerns and practices in their host country.

Cultural and religious beliefs about disease and health have a considerable influence on people’s health behaviors. We found that religious and cultural beliefs affected women’s views on cancer (seeing it as a “death sentence”), willingness to disclose family history of cancer, emphasis on treatment rather than prevention, which has been reported in other studies [40–46], and cultural taboo surrounding female sexuality and sexual activity. Most cases of cancer that women narrated ended in demise and the occurrence of survival was almost nominal. Similar beliefs about cancer have been reported in other studies with Africans, and West and South Asians [29, 32, 44–46]. All of these beliefs seem to be associated with a general lack of knowledge about screening. To address these beliefs, and increase knowledge, cervical cancer screening information needs to be framed and communicated in a non-threatening and culturally sensitive manner to mitigate the fear of cancer which appears to be one of the major impediments to undertaking cervical cancer screening for immigrant women who are already afraid of this disease. For instance, women’s fear of cancer and belief that even thinking about cancer may bring bad luck to themselves or family could be reduced by the use of non-fear-provoking cancer messages that place more emphasis on survival rather than death from cervical cancer through inclusion of stories of cervical cancer survival, (particularly of minority women whose cancer was successfully detected through screening and treated). Such messaging has the potential to reduce fear, alter the belief that thinking about cancer may cause the disease, and motivate women to participate in screening practices [47–49].

We also found reluctance in Muslim community to share information about their family history of cancer with their children. This appears to be related to their cultural beliefs and concerns for not wanting to burden children with bad news. However, this lack of knowledge regarding family history of cancer, may inadvertently devalue the importance of undertaking preventive measures due to false reassurance. Furthermore, the majority of participants discussed the differences in the health care system between their home and host countries. Participants from both ethnic groups discussed that the lack of universal health care in their home countries and the cost of medical care that they had to incur in their home countries, encouraged them more toward treatment than prevention of the disease. In addition, they reported having few screening programs or policies for early detection of chronic diseases back home. Research shows that emphasis on treatment of symptoms and anomalies in the home countries may hinder immigrant women to seek medical care while asymptomatic post migration [47, 50–52]. Our study participants stated that they often accessed specialists for their medical conditions through self-referral which is quite different from the Canadian health care system which requires referral to specialist be made by their attending physicians. These insights can be used to guide the design and implementation of culturally and linguistically appropriate cervical cancer screening education and programs to address their pre-existing beliefs about cervical cancer and screening. This also highlight the need for education about the Canadian health care system and preventive services at the time of entry to Canada, perhaps through Immigration and Settlement Services, in order to expedite access to screening services among this population.

Our findings suggest that physicians need to play an active role in education. Some participants reported that their physician did not provide them with sexual health information including about cervical cancer and screening. This is of particular concern as they also expressed the importance of receiving information and recommendation from their attending physicians as they viewed them as trusted, knowledgeable and authoritative figures. Several studies have also identified the lack of physician recommendation as a major barrier to accessing screening services [44, 53, 54]. The discussion of cancer screening in health encounters is imperative for this population considering the health care system differences, lack/limited knowledge of Muslim women about importance of screening, and their cultural beliefs surrounding the deadliness. The failure in health communication regarding cancer screenings indicates the need for health care professionals, including physicians, to be educated about the sexual health needs of Muslim immigrant clients, to provide information opportunistically during health encounters, and to notify women when they are due for their annual check-up. As younger and unmarried participants stated that wearing Hijabs and being Muslim promoted preconceived assumptions about Muslim women’s sexual lives, primary care providers also need to ensure that they provide equitable access to sexual health information and services.

Women’s religious affiliation and cultural upbringing which valued modesty and privacy hindered their ability for open dialogue about issues related to sexuality and sexual activity and made it made it difficult to visit male physicians. There was a general preference among participants to have female physicians and specialists. Preference for female physicians by Muslim women is well documented in the literature [18, 21, 42, 43]. Furthermore, many women preferred physicians who spoke their language as they find it cumbersome to explain complex health problems like sexual health in English. This result corroborates findings from studies that have found benefits (e.g., improved quality of care, satisfaction and continuance with care) in patient-physician ethnic and linguistic concordance during health encounters [55, 56]. Importantly, we found that although having a family physician with similar ethnic and linguistic background was preferred it did not always guarantee access to sexual health services or information.

Access to physicians was also a barrier to screening and prevention. Apparently, based on participants’ comments, the government’s Health Care Connect program only provides people with physicians who accept new patients in their neighborhood and does not consider people’s preferences such as sex, language, or ethnicity of primary care provider. Furthermore, several women complained about long wait time, sometime several hours, to see their attending physician regardless of having booked their appointments in advance and being forced to only discuss one health problem per visit. Long waits sometimes resulted in women going to the emergency departments or walk in clinics. This means lost opportunities to discuss preventive measures like cancer screening with Muslim immigrant women who lack the knowledge of undertaking health promotion practices and who are less familiar with Canadian health care system. This also reinforces the use of health services only for treatment rather than prevention of disease.

A strategy which may help to address this issue and reduce waiting time may be to promote enrollment in Ontario’s relatively new primary care patient enrolment models (PEM), such as Family Health Networks (FHN), Family Health Organizations (FHO) and Family Health Teams (FHT), where there is more of a focus on providing preventive care through an interdisciplinary team which often includes nurse practitioners and other health professionals. Studies have shown a higher rate of cervical and breast cancer screening for immigrant women who are enrolled in PEMs (11–13).

The study revealed that Muslim women had a positive attitude toward HPV self-sampling method. After the demonstration of the HPV self-sampling kit, many of the participants agreed that this would be a feasible and culturally acceptable strategy for Muslim women to undertake cervical screening and they would be willing to try using the kit. They believed that the proposed strategy addressed many of their concerns and discomforts about cervical cancer screening, mainly issues surrounding privacy and modesty. They stated that this strategy would allow them the opportunity to conduct the test in the privacy of their own home and at the time that is convenient as it would not interfere with work or regular home routines of the women. Moreover, they elaborated on how this approach will save them time and money as they did not have to take time off from work or pay for commuting. These are some of the barriers that have been identified in the literature [13, 16, 18–20, 44] and HPV self-sampling has the potential to overcome. Our findings supported earlier studies that explored attitudes of women towards HPV self-sampling [22–27].

Women’s proficiency and accuracy in taking the HPV samples was one of the concern raised. There were differences in views with younger women feeling more confident in conducting the test themselves whereas the older women were more hesitant. Women suggested that instruction manuals in their own language or a short vide could help them to do the test appropriately.

HPV self-sampling was preferred to other outreach methods that target never or under-screened women such as the mobile bus which the majority of our participant viewed as unclean, and more relevant for minor services like blood tests and immunization, but not sensitive issues related to sexual health.

### Limitations

Our study should be considered in light of the following limitations. First, the selection/participation biases might have resulted in a non-representative sample of Muslim women, which could also compromise the transferability of the results. Second, some women may have been concerned about providing the right answers and not appearing uninformed or provided answers that they felt were in line with the interviewer’s medically trained background. However, some women shared their traditional beliefs and practices after good rapport was established. Third, all the participants could speak English and were relatively highly educated and the majority were in Canada more than 5 years. Hence views of recent and less educated Muslim immigrants with limited English fluency may not have been fully captured in this study. Finally, the small sample size prevents generalization of the results to the broader population of Muslim women. Future studies using larger samples and quantitative research partly based on the findings reported in the present studies are warranted.

## Conclusion

This study provided critical insights about the importance of religious and cultural beliefs in shaping the daily and health care experiences of Muslim women and cancer screening decisions. Our study showed the deterring impact of beliefs and health practices in home countries of Muslim immigrant women on their utilization of screening services. The need for culturally sensitive and opportunistic health communication during health encounters are crucial to enhance women’s awareness and knowledge about cervical cancer and screening. Physicians and other healthcare providers should also be educated about sexual health communication with minority women and be aware of the detrimental impact of preconceived assumptions about sexual activity of Muslim women.

Our results demonstrated that HPV self-sampling offers a favorable alternative model of care to the traditional provider administered Pap testing for Muslim immigrant women. It has the potential to empower Muslim women and lead to a behavior change, resulting in increased participation in cervical cancer screening. The HPV-self sampling also may be valuable in promotion of cervical cancer screening among other hard-to-reach communities and reducing cancer screening disparities.
